# Supplementary material for: What determines participation in sport for older adults in England: A multilevel analysis of Active Lives data
Source: PLoS One. 2024 Apr 4;19(4):e0301790. doi: 10.1371/journal.pone.0301790 (PMC10994306; doi:10.1371/journal.pone.0301790)
Supplement: Attachment — Submitted filename: Responses to Editors and Reviewers.docx [file pone.0301790.s001.docx]

Responses to Editors and Reviewers: [PONE-D-23-04952]

Dear Dr Chenette,

On behalf of my co-authors and I, thank you for taking the time to review our manuscript. Thank you for the insightful comments and suggested revisions. Please also thank the reviewers for their time and effort in reviewing the manuscript. The comments and suggested revisions provide knowledge and go a long way to improving the manuscript.

Please see an overview of our changes in the table below. Within text we have used ‘tracked changes’ to highlight any revisions to the manuscript. I hope this is clear. Each of the comments have been addressed in detail. They have been useful in improving the content and readability of the manuscript, and we thank you for the time and effort undertaken to read the manuscript. We hope that our changes have made the manuscript clearer. These change have been outlined below.

We have no preference of a handling editor, and no objections to reviewers. The authors received no specific funding for this work. There are no conflicts of competing interest within this piece of research. The Active Lives survey is a funded project by Sport England. Sport England had no role in study design, data collection and analysis, decision to publish, or preparation of the manuscript. The researchers represent The University of Essex which does not have a conflict of interest with Sport England or the UK Government. All authors have read the manuscript and feel it represents their honest work and have approved the manuscript for submission. Findings representing this research have not been published elsewhere or are not being considered for publication elsewhere.

Yours Sincerely,

Dr Andrew Brinkley PhD

Professor Gavin Sandercock PhD

Dr Ruth Lowry PhD

Dr Paul Freeman PhD

**Overview of revisions**

| **Comment/Suggestion** | **Revisions** |
| --- | --- |
| **Academic Editor/Journal Comments** |  |
| *Please ensure that your manuscript meets PLOS ONE's style requirements, including those for file naming.* | Thank you. The manuscript has been adjusted to meet the style requirements. |
| *Thank you for stating the following financial disclosure:*  *The author(s) received no specific funding for this work. The Active Lives survey is a funded project by Sport England. However, the data analysed is publicly available on the UK Data Archive. Therefore, Sport England played no role in the analysis of the data or interpretation of the findings. Sport England was not offered the right to review our methods or analysis.*  *At this time, please address the following queries:*  *a) Please clarify the sources of funding (financial or material support) for your study. List the grants or organizations that supported your study, including funding received from your institution.*  *b) State what role the funders took in the study. If the funders had no role in your study, please state: “The funders had no role in study design, data collection and analysis, decision to publish, or preparation of the manuscript.”*  *c) If any authors received a salary from any of your funders, please state which authors and which funders.*  *d) If you did not receive any funding for this study, please state: “The authors received no specific funding for this work.”*  *Please include your amended statements within your cover letter; we will change the online submission form on your behalf.* | Thank you. This has been addressed within the cover letter. |
| *Please state what role the funders took in the study. If the funders had no role, please state: "**The funders had no role in study design, data collection and analysis, decision to publish, or preparation of the manuscript."*  *If this statement is not correct you must amend it as needed.*  *Please include this amended Role of Funder statement in your cover letter; we will change the online submission form on your behalf.* | Thank you. This statement has been included within the acknowledgements. Further, the role of the funder (of the Active Lives Survey, not the manuscript) has been included within the cover letter and acknowledgements. |
| *We note that Figure 1 in your submission contain [map/satellite] images which may be copyrighted. All PLOS content is published under the Creative Commons Attribution License (CC BY 4.0), which means that the manuscript, images, and Supporting Information files will be freely available online, and any third party is permitted to access, download, copy, distribute, and use these materials in any way, even commercially, with proper attribution. For these reasons, we cannot publish previously copyrighted maps or satellite images created using proprietary data, such as Google software (Google Maps, Street View, and Earth). For more information, see our copyright guidelines: http://journals.plos.org/plosone/s/licenses-and-copyright* | Thank you. A permission email from the body (Active Partnerships) has been uploaded. We have included the required statement within Figure 1.  Note (update October): Following an email from PLOS One we have removed figure 1 from the submission. |
| **Reviewer 1 – Comments** |  |
| *A key question is whether people in England are becoming more inactive, which is alluded to in the Background para 1 line 3. One would need trend data to justify that, which should be possible to obtain from active lives analyses at least since around 2016.* | Thanks for this comment, globally we are. However, according to the Active Lives data since 2015 adults aged over 55+ years are becoming more active, the same can also be said for 75+ years. In the region of between 1-6% dependent on age. There is, however, a concerningly high level of inactivity within the country still, as highlighted in the data presented. A line outlining that participation has increased, albeit marginally has been included (see lines 87-88). |
| *Regional Active Partnerships occurred as a result of the interest by Sport England in investing in physical activity and sport following the London 2012 Olympics. However, there is no evidence in this paper whether these partnerships implemented sport promoting activities and environments well, and whether the policies were differentially implemented across different regions; the reason why this is important is because it implies that policy implementation is more than just opportunities in the built environment as they exist, but could be a range of diverse and different strategies across regions. Has that engagement occurred, and has sport participation changed in relation to those regional partnerships? This is a fundamental underpinning concept for this paper which would need both policy analysis and further investigation of additional active lives datasets from other years. Given a decade of this policy implementation, a critical question is whether sport participation has increased, and whether the active partnerships have contributed to that. Otherwise, it undersells the relevance of active partnerships in this specific analysis.* | Thank you for these comments. It was not the purpose of the study to observe the impact of series of policies and strategies on behaviour and health over time. Rather, we sought to understand the most recent picture within the active lives data. Observing variation over time due to policy implementation is an interesting idea, and something we note within our limitations/future directions section. However, the extent in which the active lives survey is sensitive to observe these changes is debatable on the regional policy level. For example, the survey does not nest participants into their postcode/local authority. Therefore, even if it were known when, how, and where a policy or strategy had been implemented across a region, it would be tricky to observe any modifications in behaviour. With this said, the points made on policy, and how these vary in terms of implementation are insightful. These comments improve our background to sports promotion across England. We, therefore, have made multiple modifications to the background and discussion sections. |
| *The study refers to determinants, but the term correlates should be used throughout.* | Thank you. This is a useful addition and has been amended throughout. |
| *The theoretical framework here, COM-B is increasingly used in understanding physical activity participation and is likely to be relevant to sport participation, although this framework may have somewhat less relevance for older adults.* | Thank you for this comment. COM-B is a meta-theory conceptualised from multiple behavioural theories. The model is widely used by Sport England across all populations. COM-B is not an age specific model and has widely been adopted within older populations such as those cited within our study, but also within clinical and public health contexts. |
| *There is no rationale for the cutpoint of 3360 minutes per day, which is eight hours per day, which is quite unrealistic as an upper limit for truncation or winsorisation, even though it is referenced in reference number 33. Further thought on the nature of realistic cutpoints for older adults should be considered.* | Thanks for this comment. Our approach to cleaning the day is consistent with other physical activity cross-sectional studies, and those analysing active lives data. For example, Brainard et al (<https://doi.org/10.1016/j.ypmed.2019.03.043>) adopted the same cut-off threshold. |
| *There is good classification of sports into those that are moderate and vigourous, but they should be listed at least in supplementary online material.* | Thank you for this suggestion. We would be more than happy to provide this information as supplementary material, however, (i) the active lives survey requires a participant if their participation was moderate/vigorous, rather than which sports are considered moderate or vigorous. (ii) We have already signposted the reader to the UK archive page. This provides an extensive overview of the survey, including a data dictionary and codebook. |
| *One question regarding the 28-day prevalence in the active lives survey is whether seasonal variation might not influence this, or is the active lives survey a rolling continuous data collection?* | Thanks for this comment. This was not clear currently in the manuscript. Active Lives is delivered in monthly waves (12-per-year). The purpose of this, is to account for seasonal variation. Whilst we have already steered the reader to the sampling procedure within the survey, we have added a point on the sampling waves and seasonal variation within the manuscript. See lines 276-277. |
| *The measures from the active living survey will describe, but are often single item is used here to reflect call underlying psychological and psychosocial dimensions. It would be useful to have some psychometric work undertaken on these data, rather than just referencing the published scales or other work on the dimensions from which these items were developed.* | Thank you for these suggestions. The single-items used in the Active Lives Survey are a challenge. This has been acknowledged within the manuscript. Given the single-item nature of the measures, psychometric testing is limited. Indeed, one option may have been to correlate the single-items against a multiple-item measure reported by the sample, but not included presently within the study. However, to our knowledge the Active Lives survey contains no multi-item measures. Which not examining the psychometric properties of the measures, IPSOS Mori do pilot the measures prior to delivery. Please see our links to the UK data Archive. Further, our approach is consistent with previous work examine Active Lives data such as, Brainard et al. 2017, 2020; Strain et al. 2022; and Sandercock et al, 2022. Finally, we are certainly open to suggestions or solutions to address this challenge within the data. |
| *The multilevel analysis seems appropriate, but is often quite complicated, such as in table 2 with the correlation matrix of 20 variables. Given the very large sample size although most of the correlations are <0.1, they are all highly significant, an artefact of sample size.* | Thank you for this comment. At the present sample size, almost all associations will be become significant on the individual-level. This is a challenge for this form of analysis in this dataset. The correlation table should be interpreted using effect size, rather than noting statistically significant association. To assist the reader, only significant associations >.001 have been reported. |
| *The analysis describes the average moderate equivalent minutes as being 68 minutes per week for sport participation. First, the variance around this estimate is extremely high, being at least 3 1/2 times the median value. Second, is this a realistic meaning for older adults? It depends on the kinds of activities that are included in sport. For example if this is organised sport, then this is a non-credible mean value for the population older adults, but if it includes walking it could be plausible as an estimate. Given the high variance, most regions have very large lower and upper bound limits, and this does somewhat attenuate regional differences.* | Based on the Active Lives definition of ‘sport’, 68 minutes appears to be a realistic unadjusted value. As we have noted, sport, does not simply refer to organised moderate-vigorous traditional or individuals sports, but rather encompasses a range of activities with rules, structures, and traditions. Many of which are completed at a lower-intensity and for a high duration (see active lives code book). We selected this definition of sport, as this is better representative of Sport England and UK government policy. Further, the breadth of this definition and sports included, perhaps explain the wide variation in sports participation reported in our study. Indeed, some participants within the data reported low durations of high-intensity traditional sport, and others high durations of low-intensity sport (e.g., golf). |
| *Figure 1, the heat map shows that data in north-western England in Cumbria report more sport minutes, as do other regions in Northumberland and Yorkshire. The inverse is true of the south-east, where five fewer minutes are reported in Kent, Sussex, Cambridge or Hampshire. This is the inverse of physical activity that usually shows a socio-economic gradient where the south and south-east of England are more active than the more socially disadvantaged North, and reasons why these data shows such unexpected inverse gradients by deprivation should be explored.* | Thank you for this observation. On the individual-level, we did find multiple-deprivation to be a predictor of participation. This has been discussed within this manuscript. When examining the unadjusted mean data. There are several observations, we have drawn the reader’s attention to, which may explain this finding. Foremost, in our commentary on opportunity, we have noted the importance of ‘free’ to access opportunities to participate in sport (e.g., natural settings). Given the rural nature of the counties reporting greater levels of sport, and the broad definition adopted within active lives, this is one plausible definition. Further, it should be noted, in, every county, and more so rural counties (such as some of those highlighted), there is wide variation in equalities, where some households live in the least, while other the most deprived areas of England. Therefore, the adjusted data which accounts for this variation is likely more useful to a reader. |
| *Table 3 accounts for regional variation and assesses the correlates of sport, finding that factors such as age are strongly correlated. Surprisingly, and contrary to the majority of published research, gender differences in sport participation, differences by disability status and differences by body mass index appear non-significant. These unexpected correlates, or lack thereof require careful explanation in either the model all measures used. This could also explain why capability or motivation are not significant here, as in this could be a measurement issue.* | Thank you for these comments. Yes, these are interesting observations. Consistent with most papers where large-scale models are analysed, we neglected to include a detailed discussion of each of the non-significant correlates of behaviour. Studies which analyse active lives data present a mixed picture on the role of age, gender, body composition, disability status, and other intrapersonal predictors. With this said, to prompt further research, we have highlighted these observations within the discussion. |
| *On page 16 the authors mention the Covid19 influence on the results. This is important, as Covid undoubtedly influenced both individual and environment level correlates, capacities for sport participation and individual motivation for being active. It would be important to re-analyse and present data for another year, ideally pre Covid, to assess the same correlates persist . This is really important in terms of the generalisability of these correlates, as physical activities that people could do and sports that people could participate in were very different during the pandemic. As shown by Strain et al 2022 in the reference list, analyses of the active lives data over five years shows that physical activity and sport declined especially driven by 2020 restrictions. Since these restrictions overlap with the period study in this analysis, it is really important to see whether these correlates remained consistent at other time points.* | Thank you for this suggestion. Our data are cross-sectional in nature and therefore this study is not able to provide valid commentary on unique (once in a life-time) longitudinal variations in activity due to the pandemic and lockdown restrictions. We have acknowledged this as a limitation in our manuscript. The design and purpose of our multilevel analysis was to identify regional variations in sports participation with a focus on policy-level decisions and actions. The interaction between policies and changes in activity due to unforeseen circumstances such as lockdown restrictions is undoubtedly a novel and rich research idea. However, the active lives survey methodology does not have the sensitive to unpick these data on a regional- and local-level over time. Further,  a longitudinal multi-level model including both variation on the regional level and over time would be comprehensively complicated, and while we agree this might be an important next step to take, this was not the aim of our paper. However, we have noted this as a standalone point within our conclusion. |
| Finally, the policy relevance and the innovation of these findings is not clearly discussed. | Thank you for these comments. We have added some additional commentary on current policy and its implication to our findings throughout the discussion. |
| **Reviewer 2 Comments** |  |
| *It needs to be made clear in several places that this study is specific to England.* | Thank you. Reference to England has been made throughout. |
| *A Bonferroni adjusted p value is advised given the sample size and number of tests being run.* | Thank you for this comment. This has been addressed in the case of the correlations. |
| *The COVID 19 pandemic is reduced to a limitation at the end. This is important contextual information and needs to be given much more attention within the manuscript.* | Thank you for this observation. This has been expanded on. Please also see the notes on Active Live sampling, and weighting. |
| *There are a lot of grammatical errors throughout and re-wording needed in many places. Please undertake a thorough proof read. Please provide line numbers in future. Otherwise, it makes a reviewer’s job much harder.* | Thank you. A thorough proofread has been undertaken. Line numbers have been included. |
| **Abstract** |  |
| *Could a definition/description of “Active Partnerships” be offered in the abstract, especially to aid an international reader?* | Thank you. A short definition has been provided. Please see line 50. |
| *The abstract needs to make the context of the dataset clear. At present, it’s not clear which jurisdiction the dataset is drawn from.* | Thank you for highlighting this. English has replaced nationwide on line 51. |
| *There are multiple typing and punctuation errors in the abstract – please proof read thoroughly (determinants, addresses, missing comma after this in L5). There are also grammatical errors (e.g., Our findings indicate THAT when….).* | Thank you, these errors have been addressed. |
| *More information is needed on the dataset. For example, how large was the sample? Likewise, these data were collected during the COVID-19 pandemic. Some recognition of this is needed especially as this is likely to have impacted the findings. For example, older adults, the group the authors pick out later, were more impacted by lockdown restrictions than other groups.* | Thank you. Additional details of the base sample of the Active Lives Survey, and our sample has been included. See lines 52-55. |
| *It’s not clear until towards the end that older adults are the focus. This needs to be clear from the outset.* | Thank you. This has been made clear throughout the abstract. |
| *Some switching throughout the manuscript between aging and ageing.* | Thank you for highlighting this. Ageing has been used consistently within text. |
| **Main text** |  |
| *P3, para 2: is the “remain” needed? This suggests that if all these issues are addressed, SE will have no responsibly for this.* | Thank you. This has been adjusted to ‘are’. See line 96. |
| *P3: “which envisaged to “inspire a generation” could benefit from rewording and consideration of grammar rules for punctuation of which. See which again on P4.* | Thank you, this sentence has been adjusted. See page 4, lines 71-75. |
| *P3, comma needed after movement.* | Thank you, this has been included. |
| *P4, Para 2, again a comma missing – after “long-term”. Please undertake a thorough read of the document to catch these.* | Thank you, this has been included. Further, a proof-read has been undertaken. |
| *P4, Para 2, at the end of this paragraph, I am wondering: what is the key message for this paragraph? A summary sentence would be useful.* | Thank you. A summary sentence/rationale has been included. See line 145-147. |
| *P4, Para 3: determinants being re-stated is repetitive. I also could not help but feel that “and is” is needed to break this sentence up.*  *I also suggest there is some rewording needed in how the COM-B elements are described. The first two come across as categorical variables based on how they are worded, whereas the third (which I prefer) suggests it’s continuous (i.e., “extent”).* | Thank you, the repetition has been adjusted throughout. Further, the section introducing COM-B has been reworked. See line 160-171. |
| *“Though influenced by social and environmental factors such as an extrinsic stimulus capability and motivation to be individual-level predictors of behaviour(27). Whilst, opportunity varies according to social support, subjective norms, and environmental factors such as the availability of facilities and/or social actors to support participation(27)”. These are not full sentences at present and could benefit from grammar checks.* | Thank you. Agreed, this section was not clear. This has been amended throughout. |
| *P5: “As such, opportunity can, in theory, be modulated to the greatest extent by Active Partnerships”. This seems like quite a strong claim here. Couldn’t there be other policymakers with roles that are important too? A tempering of the language here would be beneficial.* | Thank you for highlighting this point. We have moderated the tone used, and included additional stakeholders, policymakers, and deliverers. Please see lines 168-171 |
| *P5: I find it hard to agree that exergaming is a form of sport (see definitions of sport elsewhere; dictionary of sport etc.). Can the authors justify this?* | To some extent, we do agree. However, this is a larger philosophical debate which was not specifically the purpose of our research. Considering the mixed definitions of what sport is, and is not, it could be argued that exergaming or e-sport meets said definition of sport, in that it can be informal/formal, includes defined rules and structures (dependent on the mode, clearly), and additional is often competitive. Further, exergaming/esport is one of the classified sports within the Active Lives survey. Given, exergaming appears to have some application when promoting sport to older adults (<https://doi.org/10.1177/0898264314551171>), and the study mentioned in text included COM-B, we feel it is useful to include for our reader. |
| *P5; in the middle of the page, which should be that.* | Thank you, this has been addressed. |
| *P5: should not-population be non-population?* | Thank you. This has been amended. |
| *P5: should “small” be included before qualitative or something like this? Otherwise, a hierarchy of evidence is being set.* | Thank you. This has been amended. |
| *Aim paragraph: similar to the abstract, please make the geographic location of this work clear.* | Thank you. The terms ‘English’ and ‘England’ have been included. |
| *“These data report during the COVID-19 pandemic” needs rewording.* | Thank you. This has been amended. |
| *P6, should “miss coded” be “miscoded”* | Thank you for highlighting this error. |
| *P6, why was 60 selected as the marker of older adult? In the introduction, 55 was the lower end of the range cited.* | Thank you for this observation. Within the background section, the data is sought from the official analysis of the Active Lives survey data and its interactive data analysis tool. The analysis groups physical activity participation into the stated age clusters (lower bound >55 years). Without downloading and analysing the data at this point of the manuscript, there is no means to provide a defined physical activity value for >60 years-65 years. The definition provided on page 6 is based upon the WHO definition of an older adult, which the threshold we set for our study. |
| *P7, explain who IPSOS MORI are (within parenthesis even) for an international reader.* | Thank you. This has been included at the first mention of the Active Lives Survey delivery. |
| *P7, the definition of sport provided here comes too late. It needs to be in the introduction.* | Thank you for this suggestion. The definition refers to what ‘sports participation’ is considered within the Active Lives survey. With this said, including the definition above adds clarity within the manuscript. |
| *P7, hyphens are necessary for 28-days and 4-weeks (four weeks).* | Thank you. Hyphens are already included within this sentence. This is consistent across the manuscript. |
| *P7: as currently written “The Active Lives survey requested participants to provide data on the number of days they participated in 200 modes” gives the impression they had to have participated in all 200 forms are currently worded. Re-wording needed.* | Thank you, this has been amended. |
| *P7, commas needed before and after “including” and Archive) in the middle of this page when referring to the survey.* | Thank you. Commas have been included. |
| *P7, “Moderate Equivalent Minutes (MEMS) spent participating in traditional modes of sport was extracted from the data.” Review whether plural or singular are used correctly here.* | Thank you, this has been reviewed. |
| *P7, “MEMS traditional team sport is constructed of activities representing moderate (MITS) and vigorous intensity traditional sports (VITS) into a composite variable.” Grammar seems off here. Please reword for reader.* | Thank you, these points have been addressed within the modifications outlined below. |
| *The word “construct” confused me and I still don’t fully understand the meaning here. Overall, the explanation from “For the purpose of the present study” to the end of this paragraph could be much better. A link could also be provided direct to the UK Data Archive page.* | Thank you for highlighting the confusion within this section. This has been amended. Further, a link to the UK Data Archive has been included. |
| *P8, there is a parenthesis within a parenthesis unnecessarily in the middle of the page.* | Thank you. We believe this error has now been amended. |
| *P8, it seems somewhat of a stretch to suggest the question for “individual development” is either reflective of psychological wellbeing or individual development. It seems to be a measure, to some degree, of generalised self-efficacy.* | Thank you for this comment. Originally, we came to the same conclusion, regarding the content of the measure. However, we opted the adopt the term used in the Active Lives Survey user guides, and materials. With this said, we feel self-efficacy is better reflective of the measure and this has been adjusted throughout the manuscript. |
| *P8, should active partnerships have capital letters near the bottom?* | Thank you for highlighting this error. Active Partnerships is now consistently listed throughout the text. |
| *P9; no need for Foremost. Do you mean first?* | Thank you, ‘foremost’ has been removed. |
| *One thing I would ask about is were the information regarding when the data were collected? It seems important that some data were collected during lockdown periods. Was this controlled for?* | Thank you. The data is collected in twelve moderately weighted waves of data collection (n=500 participants per region), where in the region 50,000 participants are sampled per wave. The purpose of this, is to account for seasonal variation, or other major events influencing behaviour, such as the social restrictions influencing behaviour. Given, the ultimately equal application of social restrictions across the UK in late 2020-2021, and lack of the ability to unpick the time and state of completion of the survey (e.g., when tiered restrictions were applied to regions), we are happy with the equally weighted sampling with the Active Lives Survey. With this said, we have added additional commentary on the role of the pandemic across the paper. Further, we have additional considerations with the limitations section. |
| *Given the sample size and the number of variables, a Bonferroni adjusted p value should be used. The correlation table is showing that even r = .02 is significant, yet that is really a negligible relationship.* | Thank you for this comment. This is a challenge for this form of analysis in this dataset. The correlation table should be interpreted using effect size, rather than noting statistically significant association. To assist the reader, only significant associations >.001 have been reported. |
| *Table 1: the n for each Active Partnership would be useful.* | Thank you for highlighting this. Samples within each Active Partnership have been included. |
| *Table 3: some p values are 2 decimals, and others 3.* | Thank you for highlighting this error. P-values are now reported with three decimal places. |
| *P15: “national population representative picture” either needs punctuation or needs to be more concise. This sentence also needs to state the location. The English focus of the study also needs to come through in subsequent sentences.* | Thank you this has been amended. Further, consistent with changes above, emphasis has been placed on the English focus of the study throughout the discussion. |
| *P15: “29% of the recommended weekly guidelines” – be clear that these are recommended PA guidelines, not sport.* | Thank you, this was not clear. The phrase ‘physical activity’ has been included. |
| *P15: the data are cross-sectional, so the idea of prediction cannot be substantiated by the dataset.* | Thank you. This has been modified throughout. |
| *P15: the “, which” in the second line of para 2 should be that.* | Thank you, this has been addressed. |
| *P15: this finding that COM-B elements are not working is consistent with lots of research in exercise psychology pointing to the limits of such social-cognitive models (see Ekkekakis).* | Thank you. This is a fair comment on the limitations of such models, or indeed how the factors within are measured. In the case of our study, it may be the measures assessing capability and motivation are somewhat limited. This has been acknowledged within the manuscript. |
| *While it is being suggested that better sport options are needed, do older adults actually want sport in these areas? There is great room here to point towards the need to work with older adults in these communities to establish what works.* | Thank you, agreed. We have already placed some emphasis on adopting participatory approaches with older-adults across regions/places. We have now included additional commentary on the importance of understanding the experiences, attitudes and needs of older adults across the discussion. |
| *P16: “and places have been associated with the basic need relatedness, belonging, an identity, and longevity in sport” – this needs to be reworded.* | Thank you. This has been reworded. |
| *P16: unnecessary parenthesis after accessible.* | Thank you for highlighting this error. This has been removed. |
| *P16, “whether social or physical”- a comma is needed before and after this segment.* | Thank you. This sentence has been adjusted. |
| *P17, comma needed before “An area”.* | Thank you. This sentence has been adjusted. |
| *P17: should perceptions of opportunity be modified solely? What about changing actual opportunity? This comes later, but surely should be coming first.* | Thank you. This has been adjusted. |
| *P17: “participatory placed based approaches” needs several pieces of punctuation.* | Thank you. This sentence has been adjusted. |
| *P17: socially enriching should be “socially-enriching,”…* | Thank you, this has been adjusted to socially-enriching. |
